# Prognostic performance of clinical assessment tools following hip fracture in patients with chronic kidney disease

**DOI:** 10.1007/s11255-021-02798-7

**Published:** 2021-03-08

**Authors:** Henry H. L. Wu, Reinier Van Mierlo, George McLauchlan, Kirsty Challen, Sandip Mitra, Ajay P. Dhaygude, Andrew C. Nixon

**Affiliations:** 1 Department of Renal Medicine, Lancashire Teaching Hospitals NHS Foundation Trust, Royal Preston Hospital, Sharoe Green Lane, Preston, PR2 9HT UK; 2 Faculty of Medical and Human Sciences, University of Manchester, Manchester, UK; 3 Department of Physiotherapy, Lancashire Teaching Hospitals NHS Foundation Trust, Royal Preston Hospital, Preston, UK; 4 Department of Orthopedics & Traumatology, Lancashire Teaching Hospitals NHS Foundation Trust, Royal Preston Hospital, Preston, UK; 5 Department of Accident and Emergency, Lancashire Teaching Hospitals NHS Foundation Trust, Royal Preston Hospital, Preston, UK; 6 Manchester Academy of Health Sciences (MAHSC), Manchester University NHS Foundation Trust, Manchester, UK; 7 Department of Renal Medicine, Manchester University NHS Foundation Trust, Manchester, UK; 8 National Institute of Health Research D4D MedTech and In Vitro diagnostics Co-operatives (MICs), Sheffield, UK

**Keywords:** Chronic kidney disease, Hip fracture, Geriatric nephrology, Frailty, Mortality, Prognosis

## Abstract

**Purpose:**

People living with chronic kidney disease (CKD) are at a higher risk of hip fracture with an associated increased mortality risk compared to individuals without CKD. Our study aimed to evaluate the clinical assessment tools that best predict mortality risk following hip fracture for patients with CKD.

**Methods:**

Patients with CKD G3b-5D admitted to Lancashire Teaching Hospitals NHS Foundation Trust, U.K. between June 2013 and Dec 2019 were included. The association between CKD and post-fracture mortality risk was evaluated. All patients were assessed using tools that evaluated frailty status, co-morbidity, pre-operative risk, functional status and cardiopulmonary fitness. Receiver operating characteristic curve analyses were performed to determine the prognostic accuracy of the assessment tools for 30 day and 1 year mortality following hip fracture in patients with CKD.

**Results:**

397 patients fulfilled inclusion criteria with a mean age of 83.5 ± 9.2 years. Older age, female sex, intracapsular fracture and more severe CKD, co-morbidity and frailty status were all associated with an increased mortality risk. Patients with dialysis-dependent CKD and severe/very severe frailty had a hazard ratio for mortality of 2.55 (95% Cl 2.11–2.98) and 3.11 (95% Cl 2.47–3.93), respectively. The Clinical Frailty Scale demonstrated the best prognostic accuracy for both 30 day [Area Under the Curve (AUC) 0.91, 95% Cl 0.84–0.97] and 1 year mortality (AUC 0.93, 95% Cl 0.87–1.00).

**Conclusion:**

Patients with advanced CKD and severe frailty have a high mortality risk following hip fracture. The Clinical Frailty Scale is an excellent prognostic tool for mortality in this setting and could be easily incorporated into routine clinical practice.

## Introduction

Hip fracture presentations are a global public health burden [1]. There were an estimated 1.6 million hip fractures in 2000; this is projected to increase to 6.3 million by 2050 with a growing elderly population [2]. People living with chronic kidney disease (CKD) have a greater risk of falls leading to hip fracture [3]. Individuals with end-stage kidney disease (ESKD) are reported to have a risk of falls and hip fracture between 4 and 14 times greater than those without CKD [4, 5]. There are higher risks of poor health outcomes for patients living with CKD after sustaining hip fracture. Increased morbidity and mortality following hip fracture is compounded by factors such as frailty [6], sarcopenia [7] and CKD-associated mineral and bone disorder (CKD-MBD) [8]. A significant decline in functional status following acute trauma is frequently observed for patients with various co-morbidities including CKD [9]. Due to this marked decline, rehabilitation following hip fracture is more difficult for patients with CKD compared to the general population [9]. Importantly, mortality outcomes are worse for patients with reduced functional status [10].

Though multiple risk factors are suggested, the most important predictors of mortality risk following hip fracture are unclear for patients living with CKD. Tools prognosticating mortality risk following hip fracture have been previously suggested and are widely applied in practice for the general population [11–13]. However, these tools have not been validated for patients who have CKD. Risk stratification tools that guide clinical decision-making may optimize outcomes in CKD populations after acute trauma. Our study aims to evaluate risk factors for mortality and the relative prognostic accuracy of various clinical assessment tools following hip fracture for patients living with CKD.

## Methods

### Study design and participant selection

Patients with non-dialysis and dialysis-dependent CKD (G3b-5D) admitted to Lancashire Teaching Hospitals NHS Foundation Trust with a hip fracture were included in this study. Our study was a secondary analysis of a larger prospective cohort study, which investigated mortality outcomes in the general population admitted with hip fracture.

Data were collected between June 2013 and December 2019. Formal patient consent was not required, because data were collected as part of routine clinical practice. Ethical approval was obtained from the North West Health Research Council, UK and the NHS Health Research Authority.

### Data collection

Demographic and clinical characteristic data including age, sex, CKD stage, living arrangements and type of hip fracture sustained were recorded within 48 h of hospital admission. Mortality from the date of hospital admission was recorded. Clinical assessment tools evaluated in our study were categorized into frailty, co-morbidity, pre-operative risk, functional status and cardiopulmonary fitness assessment tools.

#### Frailty

The Clinical Frailty Scale (CFS) [14] is an ordinal frailty assessment tool that provides nine descriptions of levels of fitness or frailty. It relies upon a health professional’s assessment of an individual’s frailty status. Individuals with a CFS score of 1–2 are considered fit or well [15]. A CFS score of 3–4 suggests individuals may have a health problem but managing well, though not regularly active, or they may be becoming vulnerable, but are not overtly frail [15]. A CFS score of 5–6 identifies those with signs of frailty but otherwise have some degree of independence [15]. Individuals with a CFS score of 7–9 have severe or very severe frailty [15].

The CKD Frailty Index Lab (CKD FI-LAB) [16] is a composite score based upon the FI-LAB in which blood pressure readings and laboratory variables are used to estimate frailty status. The FI-LAB has been studied in the general older population and is predictive of clinical outcomes [17, 18]. The CKD FI-LAB is calculated by the total number of deficits divided by the total number of variables measured. “Appendix” lists the variables included in the CKD FI-LAB scoring. A higher CKD FI-LAB score suggests greater degree of frailty.

#### Co-morbidity

Charlson’s Co-morbidity Index (CCI) [19] is a composite score of co-morbidity status that is predictive of mortality in the general and CKD population [20]. A higher CCI score represents greater co-morbidity.

#### Pre-operative risk scores

The American Society of Anesthesiologists (ASA) Index [21] is a five-category ordinal classification system used to determine pre-operative fitness for surgery. It ranges from category 1, a healthy person, to category 5, a moribound person who is not expected to survive beyond 24 h with or without surgery. The Nottingham Hip Fracture Score (NHFS) [22] is a 10-point scoring system, which aims to predict 30 day mortality risk following hip fracture. A higher NHFS score represents higher mortality risk. The Sernbo score [23] is used to predict mortality risk following hip fracture and is scored out of 20. Scoring is determined from 4 components: age, social situation, mobility and mental status. A lower Sernbo score represents higher mortality risk.

#### Functional status

The Karnofsky Performance Status (KPS) score [24] measures functional status through assessing an individual’s ability to perform everyday activities. The KPS score ranges from 0 to 100, with higher KPS scores suggesting better functional status.

#### Cardiopulmonary fitness

The Duke Activity Status Index (DASI) [25] is a 12-item self-administered questionnaire that measures functional capacity. Through the formula $$(0.43$$$$\; \times \;{\text{DASI)}}\;{ + }\;{9}{\text{.6}}$$$$\; \times \;{\text{DASI)}}\;{ + }\;{9}{\text{.6}}$$$$\; \times \;{\text{DASI)}}\;{ + }\;{9}{\text{.6}}$$, an estimated VO_2_ peak value can be calculated [26]. The estimated VO_2_ peak provides an estimation of an individual’s peak oxygen uptake and has been proven to be a reliable measure of cardiorespiratory fitness [27].

### Statistical analysis

All statistical analyses were performed on Stata 14.2 (StataCorp, College Station, TX, USA) or StatsDirect Statistical Software (version 3.2.10, 03/05/2020). Descriptive statistics summarized demographic and clinical data. Frequencies and percentages were used to present categorical data. Mean ± SD values were presented for continuous variables that were normally distributed. Otherwise, continuous data were presented as median ± IQR.

Multivariate Cox regression analysis was used to evaluate the association between CKD and post-fracture mortality risk. Independent variables were selected a priori. Age (for each 1 year increase), sex, intracapsular hip fracture, CKD stage (with CKD G3b being the reference), CCI (for each unit of increase) and CFS score (with CFS 1–2 being the reference category). The assumption of proportional hazards was assessed by reviewing the significance of time-variable interactions.

An area under the curve (AUC) value was calculated through receiver operating characteristic (ROC) curve analyses to assess each assessment tool’s prognostic value for 30 day and 1 year mortality. A two-tailed *p* value < 0.05 was considered statistically significant. As a secondary analysis, there was no a priori sample size calculation.

## Results

**Fig. 1:**
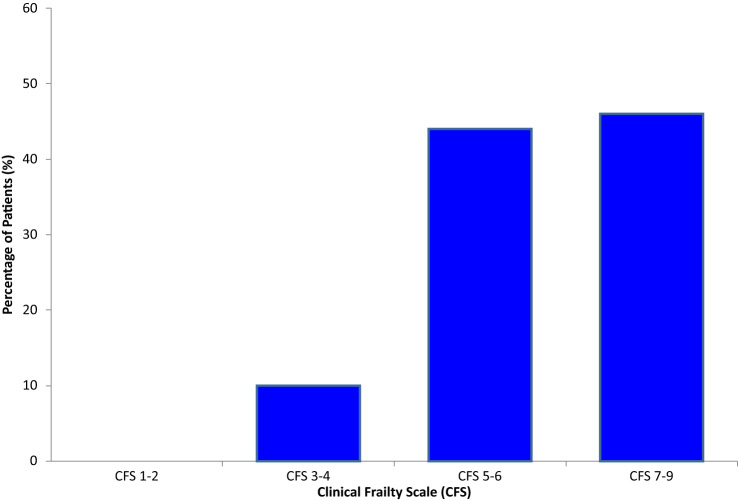
Distribution of CFS scores amongst patients living with CKD admitted with hip fracture. This figure was created from StatsDirect Statistical Software (version 3.2.10, 03/05/2020)

Amongst 2743 patients hospitalized following hip fracture between June 2013 and December 2019, there were 397 patients living with CKD G3b-5D. Table 1 demonstrates the demographic and clinical characteristics of patients with CKD G3b-5D in this study. The mean age was 83.5 ± 9.2 years and 63% were female. Two hundred and fifty-three patients (64%) had CKD G3b. There were 42 patients (11%) receiving long-term dialysis (CKD G5D). Two hundred and twenty-five patients (57%) were admitted from home with the remaining patients admitted either from nursing or residential home. One hundred and eighty-three patients (46%) had a CFS 7–9 score. Figure 1 describes the distribution of CFS scores observed. Two hundred and one patients (51%) sustained a displaced, intracapsular fracture. One hundred and sixty-one patients (41%) received a hemiarthroplasty and 127 patients (32%) were treated with a dynamic hip screw procedure. Other observed treatment modalities for hip fracture included total hip replacement, intramedullary nail insertion, cannulated hip screw insertion and conservative management.

**Table 1:** Demographic and clinical characteristics amongst patients living with CKD admitted with hip fracture

Demographic and clinical characteristics	Patients with CKD 3b-5D (*n* = 397)
Age in years, mean ± SD	83.5 ± 9.2
Female (%)	63
CKD Stage, *n* (%)	
G3b	253 (64)
G4	65 (16)
G5	37 (9)
G5D	42 (11)
Body mass index, mean ± SD	23 ± 6.1
Blood pressure in mmHg, mean ± SD	
Systolic	141 ± 17.3
Diastolic	82 ± 11.1
Hemoglobin in g/dL, mean ± SD	11.4 ± 1.2
Serum creatinine in μmol/L, mean ± SD	172.0 ± 140.3
Serum albumin in g/dL, mean ± SD	3.7 ± 0.6
Living arrangements, *n* (%)	
Home	225 (57)
Residential home	23 (5)
Nursing home	149 (38)
Frailty assessment	
CFS 1–2, *n* (%)	0 (0)
CFS 3–4, *n* (%)	40 (10)
CFS 5–6, *n* (%)	174 (44)
CFS 7–9, *n* (%)	183 (46)
CKD FI-LAB, mean ± SD	0.352 ± 0.115
Co-morbidity	
CCI, mean ± SD	12.7 ± 3.3
Pre-operative risk scores	
ASA Index, median (IQR)	3 (1)
NHFS, mean ± SD	5.8 ± 1.6
Sernbo score, mean ± SD	12.8 ± 3.6
Functional status	
KPS, median (IQR)	50 (30)
Cardiopulmonary fitness	
Estimated VO_2_ peak in L/min, mean ± SD	13.9 ± 4.1
Type of hip fracture, *n* (%)	
Intracapsular undisplaced	22 (5)
Intracapsular displaced	201 (51)
Intertrochanteric	151 (38)
Subtrochanteric	11 (3)
Pathological	12 (3)

*CKD* Chronic Kidney Disease, *CFS* Clinical Frailty Scale, *CKD FI-LAB* Chronic Kidney Disease Frailty Index Lab, *CCI* Charlson’s Comorbidity Index, *ASA Index* American Society of Anesthesiologists Index, *NHFS* Nottingham Hip Fracture Score, *CFS* Clinical Frailty Scale, *KPS* Karnofsky Performance Status Score

### Risk factors for mortality following hip fracture for patients living with CKD

**Fig. 2:**
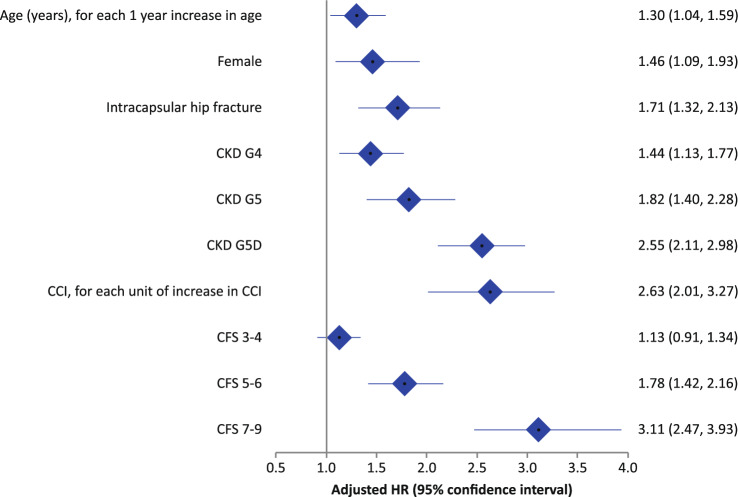
Hazard ratio forest plot of risk factors for mortality after hip fracture in patients living with CKD. *CKD* Chronic Kidney Disease, *CCI* Charlson’s Co-morbid**i**ty Index, *CFS* Clinical Frailty Scale. CKD G3b is the reference category for adjusted HR calculated for CKD G4, CKD G5 and CKD G5D. CFS 1–2 is the reference category for adjusted HR calculated for CFS 3–4, CFS 5–6 and CFS 7–9. This figure was created from StatsDirect Statistical Software (version 3.2.10, 03/05/2020)

Figure 2 illustrates a hazard ratio (HR) forest plot of risk factors for mortality following hip fracture in patients with CKD. The median follow-up was 27.2 months. The HR for each year increase in age was 1.30 (95% Cl 1.04–1.59). Female patients (HR 1.46, 95% Cl 1.09–1.93) and those who sustained an intracapsular fracture (HR 1.71, 95% Cl 1.32–2.13) had a greater mortality risk. The HR for each unit of increase in CCI score was 2.63 (95% Cl 2.01–3.27). Patients with CKD G5D (HR 2.55, 95% Cl 2.11–2.98) had higher HR compared to those with CKD G4 (HR 1.44, 95% Cl 1.13–1.77) and G5 (HR 1.82, 95% Cl 1.40–2.28). Patients scoring CFS 7–9 (HR 3.11, 95% Cl 2.47–3.93) had higher HR compared to those scoring CFS 5–6 (HR 1.78, 95% Cl 1.42–2.16) and CFS 3–4 (HR 1.13, 95% Cl 0.91–1.34).

### Prognostic accuracy of clinical assessment tools for mortality following hip fracture in patients living with CKD

Table 2 summarizes the prognostic accuracy of selected clinical assessment tools for 30 day and 1 year mortality. All 397 patients were evaluated for 30 day mortality, whilst 1 year follow-up data were only available for 299 patients. Thirty-day and 1 year mortality rates were 9.6% and 38.0%, respectively.

**Table 2:** Prognostic accuracy of clinical assessment tools for 30 day and 1 year mortality after hip fracture in patients living with CKD

	30 day mortality (*n* = 397)		1 year mortality (*n* = 299)	
	AUC value (95% Cl)	*p* value	AUC value (95% Cl)	*p* value
Frailty assessment				
CFS	0.91 (0.84–0.97)	< 0.001	0.93 (0.87–1.00)	< 0.001
CKD FI-LAB	0.78 (0.71–0.85)	< 0.001	0.83 (0.77–0.90)	< 0.001
Co-morbidity				
CCI	0.82 (0.75–0.88)	< 0.001	0.85 (0.78–0.92)	< 0.001
Pre-operative risk scores				
ASA Index	0.75 (0.69–0.81)	< 0.001	0.77 (0.70–0.84)	0.202
NHFS	0.74 (0.67–0.80)	< 0.001	0.67 (0.60–0.73)	0.357
Sernbo score	0.71 (0.64–0.78)	< 0.001	0.68 (0.61–0.74)	< 0.001
Functional status				
KPS	0.82 (0.76–0.89)	< 0.001	0.84 (0.78–0.90)	< 0.001
Cardiopulmonary fitness				
Estimated VO_2_ peak	0.69 (0.62–0.76)	0.246	0.73 (0.67–0.80)	0.512

*CCI* Charlson’s Co-morbidity Index, *CKD FI-LAB* Chronic Kidney Disease Frailty Index Lab, *CFS* Clinical Frailty Scale, *ASA Index* American Society of Anesthesiologists Index, *NHFS* Nottingham Hip Fracture Score, *KPS* Karnofsky Performance Status Score

The CFS had the best overall prognostic performance amongst the tools assessed. The CKD FI-LAB, CCI and KPS demonstrated good predictive accuracy for 30 day and 1 year mortality. AUC values for the pre-operative assessment scores, ASA index and NHFS were statistically significant for 30 day, but not for 1 year mortality. The AUC value for estimated VO_2_ peak was not statistically significant for 30 day or 1 year mortality outcomes.

## Discussion

To our knowledge, this is the first study that investigates the prognostic performance of clinical assessment tools following hip fracture for patients living with CKD. Selection of clinical assessment tools is guided by identifying the major risk factors for mortality within this context.

Similar to previous studies, older age was demonstrated to be an important risk factor for mortality following hip fracture in CKD [28, 29]. Nitsch et al. concluded that there is almost a twofold increase in hip fracture-related mortality amongst older people with an eGFR < 45 ml/min/1.73 m^2^ [28]. Postmenopausal-related changes in bone and mineral metabolism observed in older women leads to greater osteoporotic risks [30]. CKD may exacerbate osteoporotic risks to a greater extent for women compared to men due to its effects on bone and mineral metabolism [8].

There is limited data directly comparing mortality outcomes between dialysis-dependent and non-dialysis CKD groups following hip fracture. However, patients on dialysis have a higher risk of in-patient mortality following hip fracture [31–33]. Hickson et al. noted that 8% of dialysis-dependent patients who experienced a hip fracture died before hospital discharge [31]. Furthermore, there was a threefold increase in adjusted mortality risk for the dialysis-dependent group compared to patients with non-dialysis-dependent CKD [31].

Our study demonstrates that sustaining an intracapsular hip fracture is associated with an increased mortality risk for patients with CKD. The impact of having an intracapsular hip fracture from acute trauma should not be underestimated. Hemiarthroplasty, total hip replacement or internal fixation is indicated in most circumstances to treat intracapsular fractures [34]. The type of operation performed depends on multiple factors, such as hip fracture displacement, age, co-morbidity, functional status and hip joint condition prior to fracture [35]. There are patients who do not undergo total hip replacement following a displaced intracapsular hip fracture because of the surgical risks involved and no functional benefits are expected. Current evidence suggests better post-operative outcomes and lower re-operation rate from hemiarthroplasty compared to internal fixation for patients with displaced, intracapsular hip fractures [36]. CKD-MBD and delayed ability of wound healing may worsen post-operative outcomes for patients living with CKD [5, 37]. Recent evidence reported associations between post-hemiarthroplasty mortality risk and baseline renal function [38, 39]. In a case–control study of 59 patients receiving hemiarthroplasty following hip fracture, ESRD patients were more likely to develop cardiopulmonary complications and hyperparathyroidism post-operatively compared to the non-ESRD group [39]. Moreover, a greater mortality risk was observed over the study follow-up period.

Frailty was associated with an increased mortality risk following hip fracture in patients with CKD in a graded fashion. The CFS displayed excellent prognostic accuracy and had the best performance amongst the clinical assessment tools evaluated in this study. The CFS is a practical tool, taking only a few minutes to complete, and is now widely used in the acute medical setting [40]. The CFS has been shown to have good diagnostic accuracy for physical frailty in patients living with CKD [16]. Since the onset of the COVID-19 pandemic, the National Institute for Health and Care Excellence (NICE) published a COVID-19 critical care guideline that recommended the use of the CFS to inform care decisions in acute hospital admissions, including major trauma [41].

In our analysis, the CKD FI-LAB demonstrated good prognostic value for 30 day and 1 year mortality following hip fracture in patients living with CKD. A recent study suggested the CKD FI-LAB had poor diagnostic accuracy for frailty in patients living with advanced CKD [16]. Despite this finding, the FI-LAB has previously displayed excellent prognostic accuracy for mortality in the general elderly population [17, 18]. In the context of hip fracture, the CKD FI-LAB had good prognostic accuracy in patients living with CKD. The usefulness of the CFS may be limited by the experience of the assessor; the CKD FI-LAB is a more objective assessment tool and, therefore, may potentially be a more reliable alternative.

The CCI is widely used in the assessment of co-morbidity status and has good prognostic accuracy for in-hospital mortality in the general older population following hip fracture [42, 43]. Patients with higher CCI scores in addition to CKD have an associated worse health-related quality of life (HRQOL) and an increased mortality risk after fracture [44]. Post-fracture rehabilitation outcomes are worse in patients with CKD compared to those without renal impairment [44]. A multivariate-adjusted risk prediction model evaluating the predictive ability of CCI for mortality from 1990 to 2007 in the US National Hospital Discharge Survey Study displayed AUC values of up to 0.77 [42]. Multiple sources advocate the use of CCI as a cost-effective assessment tool when treatment decisions are made for the general older population following acute trauma [45, 46]. The applicability of CCI for this purpose in patients living with CKD requires further validation.

There is a significant association between functional status decline and increased mortality risk in patients living with advanced CKD [47, 48]. Our results demonstrated that admission KPS assessment had good prognostic accuracy for 30 day and 1 year mortality. Literature evaluating the prognostic value of functional status for mortality outcomes following hip fracture in CKD cohorts is limited. Nevertheless, a preliminary study from Sakabe et al. highlighted pre-fracture ambulatory status as the only prognostic indicator of life expectancy following hip fracture in dialysis-dependent patients [49].

Data evaluating the prognostic accuracy of the ASA Index, NHFS, Sernbo Score and estimated VO_2_ peak following hip fracture in patients living with CKD has not been previously reported. The ASA Index, NHFS and Sernbo Score have been shown to be useful predictors of mortality outcomes following hip fracture in the general population [50–52]. However, in our study, these clinical assessment tools were demonstrated to have only poor or fair prognostic accuracy for post-fracture mortality in patients with CKD.

Notwithstanding our study’s holistic approach and practical usefulness, there are recognized limitations. These results were collected from a single-centre with a predominantly White British population and may not be reproducible for a different patient population. Another limitation of our study is that test–retest reliability and inter-observer reliability was not assessed. A causative link between frailty, co-morbidity, functional status and mortality cannot be established due to the non-randomized controlled design of this study. Moreover, the influence of specific orthopedic interventions on post-fracture outcomes was not assessed. Research which compares outcomes of patients with CKD with hip fracture and other associated co-morbidities following different orthopedic interventions is needed.

In conclusion, patients with CKD, particularly advanced CKD, and patients with CKD and severe frailty have a high-mortality risk following hip fracture. The CFS is an excellent prognostic tool for mortality following hip fracture for patients living with CKD and could be easily incorporated into routine clinical practice. Further studies are required to evaluate interventions that aim to improve outcomes following hip fracture for patients with CKD, particularly those with CKD and living with frailty.

## Appendix

**Table 3:** The chronic kidney disease frailty index lab (CKD FI-LAB)

Variable	Lower cut off	Upper cut off
Systolic BP, mmHg	90	140
Diastolic BP, mmHg	60	90
Haemoglobin, g/L	100	120^a^
MCV, fl	82	98
White cell count, × 10^9^/L	4.0	11.0
Platelet count, × 10^9^/L	140	440
Ferritin, μg/L	100^b^	800
Transferrin saturation, %	20	50
Sodium, mmol/L	133	146
Potassium, mmol/L	3.5	5.3
Bicarbonate, mmol/L	22	29
CRP, mg/L	0	5
Corrected calcium, mmol/L	2.20	2.60
Phosphate, mmol/L	0.80	1.50
Alkaline phosphatase, U/L	30	130
Albumin, g/L	35	50
Total protein, g/L	60	80
ALT, U/L	0	41
Bilirubin, μmol/L	0	21
Prothrombin time, seconds	9	12
Fibrinogen, g/L	1.8	4.5
Folate, μg/L	3.9	19.8
TSH, mU/L	0.35	5.00
T4, pmol/L	11.0	23.0
B12, ng/L	200	900 ng/L
HbA1C, mmol/mol	20	41
Vitamin D, nmol/L	50	150
$${\text{CHD}}\;{\text{FI-LAB}}\;{ = }\;\frac{{\text{Total number of variables with deficits}}}{{\text{Total number of variables measured}}}$$		

*BP* Blood Pressure, *MCV* Mean Corpuscular Volume, *CRP* C-Reactive Protein, *ALT* Alanine Transaminase, *TSH* Thyroid-Stimulating Hormone

^a^If the individual is on Erythropoietin Stimulating Agent. Otherwise, hemoglobin upper cut off 165 g/L for women and 180 g/L for men.

^b^If the individual’s hemoglobin less than 110 g/L and/or receiving Erythropoietin Stimulating Agent. Otherwise, ferritin lower cut off 20 µg/L for men and 15 µg/L for women

Appendix is adapted from the supplementary table 4 in Nixon AC, Bampouras TM, Pendleton et al. (2019) Diagnostic Accuracy of Frailty Screening Methods in Advanced Chronic Kidney Disease. Nephron 141: 147–155, requiring permission from S. Karger AG for re-use. (Table 3)
